# Respiration resolved imaging with continuous stable state 2D acquisition using linear frequency SWEEP

**DOI:** 10.1002/mrm.27834

**Published:** 2019-06-10

**Authors:** L. H. Jackson, A. N. Price, J. Hutter, A. Ho, T. A. Roberts, P. J. Slator, J. R. Clough, M. Deprez, L. McCabe, S. J. Malik, L. Chappell, M. A. Rutherford, J. V. Hajnal

**Affiliations:** ^1^ Biomedical Engineering, School of Imaging Sciences and Biomedical Engineering Kings College London London United Kingdom; ^2^ Department of Women and Children's Health, School of Life Course Sciences King's College London London United Kingdom; ^3^ Centre for Medical Image Computing University College London London United Kingdom

**Keywords:** angiography, 4D, fetal, free‐breathing, placental, RF, steady state

## Abstract

**Purpose:**

To investigate the potential of continuous radiofrequency (RF) shifting (SWEEP) as a technique for creating densely sampled data while maintaining a stable signal state for dynamic imaging.

**Methods:**

We present a method where a continuous stable state of magnetization is swept smoothly across the anatomy of interest, creating an efficient approach to dense multiple 2D slice imaging. This is achieved by introducing a linear frequency offset to successive RF pulses shifting the excited slice by a fraction of the slice thickness with each successive repeat times (TR). Simulations and *in vivo* imaging were performed to assess how this affects the measured signal. Free breathing, respiration resolved 4D volumes in fetal/placental imaging is explored as potential application of this method.

**Results:**

The SWEEP method maintained a stable signal state over a full acquisition reducing artifacts from unstable magnetization. Simulations demonstrated that the effects of SWEEP on slice profiles was of the same order as that produced by physiological motion observed with conventional methods. Respiration resolved 4D data acquired with this method shows reduced respiration artifacts and resilience to non‐rigid and non‐cyclic motion.

**Conclusions:**

The SWEEP method is presented as a technique for improved acquisition efficiency of densely sampled short‐TR 2D sequences. Using conventional slice excitation the number of RF pulses required to enter a true steady state is excessively high when using short‐TR 2D acquisitions, SWEEP circumvents this limitation by creating a stable signal state that is preserved between slices.

## 1 INTRODUCTION

Free‐breathing three‐dimensional imaging of abdominal organs is a challenging application of MRI that requires the spatial encoding of large volumes in the presence of complex, non‐rigid motion induced by quasi‐periodic respiration^1^, ^2^ and other processes. Such motion induces ghosting and blurring artifacts that can result in non‐diagnostic images. Respiration artifacts can be avoided by imaging during a breath hold, and this is a common solution in clinical exams.^3^ However, this constrains acquisition time and can still produce motion artifacts if multiple breath holds are required or if patient compliance is compromised. The complexity of abdominal motion inhibits gated motion compensation strategies such as those used successfully for cardiac imaging as these assume that tissues return to the same position and shape within a periodic motion pattern. This condition may not be the case with abdominal imaging and is rarely the case in fetal/placental imaging. Another alternative is to perform very rapid volume acquisitions by sacrificing spatial resolution for acquisition speed, limiting their application for large abdominal volume imaging. Rapid acquisitions can be used in combination with high acceleration factors or motion tolerant non‐Cartesian k‐space trajectories with sparse reconstruction methods.^4^, ^5^ This strategy can effectively preserve image resolution but is susceptible to acquisition and reconstruction artifacts that can cause blurring or suppress texture, and the required reconstructions can be computationally expensive.

Another approach is to consider local 2D slices, for which acquisitions can be fast enough to freeze the effects of in‐plane motion. Volume coverage can be achieved using multiple 2D slice acquisitions (M2D). However, slices are likely to be geometrically inconsistent in through‐plane directions because of changes in anatomical position between individual slice acquisitions. In cases where the change is a rigid body motion or deformations are locally rigid this inconsistency can be corrected using slice‐to‐volume registration.^6^, ^7^ This approach has been used to great effect in applications where acquisition cannot be practically synchronized to motion such as the fetal brain.^8^ In the case of free‐breathing abdominal imaging it is necessary to sample a slice location at multiple points across the respiration cycle to fully encode for motion. For this purpose a dynamic series of slices is needed so that each location can be sampled in multiple respiratory states. This leads to a requirement for dense spatial and temporal 2D sampling.

Common methods for rapid slice acquisition such as single shot fast spin echo (ssFSE) and EPI readouts work best when there is a significant recovery period between shots in the same location. This creates temporally sparse sampling meaning that changes in organ shape or patient position over the acquisition duration create discontinuous data. Dense temporal sampling of nearby slices using these methods would result in saturated images, in this case steady state acquisitions using short repeat times (TR) are more effective. When TR ≪ T_2_ of tissue coherence pathways persist across multiple TRs. Applying a rapid series of radio frequency (RF) pulses initially produces complex oscillatory magnetization evolution. After a period of approximately 3 × T_1_ a steady state is achieved.^9^ It is important to avoid data acquisition during the transient period as the unstable signals can result in strong image artifacts. This is commonly controlled by a series of dummy excitations at the start of each new slice. In the case where the imaging target is moving, any motion that occurs during these startup pulses is not encoded and any information describing this motion is lost. A number of methods have been developed to shorten the duration of this transient period such as using a half‐alpha pulse^10^ or more complex catalyzation schemes.^11^ When acquiring rapid M2D slices the requirement for dummy cycles or catalyzation pulses at the start of each slice can become a significant component of the acquisition making it temporally inefficient.

In this work, we propose an excitation scheme that provides M2D volumetric coverage using a slice excitation that is continuously moving across the volume of interest, we term this method “SWEEP.” The SWEEP method shares some similarities with continuous moving table (CMT) acquisition used for extended field of view (FOV) imaging.^12^, ^13^ In CMT, the table is moved at a constant velocity that ensures that the subject moves at a rate where every phase encode step of a slice acquisition is at the scanner isocenter. However, CMT methods are limited to acquisition in the direction of table motion. Continuously shifting the slice location with each RF pulse has been proposed recently for helical time‐of‐flight (hTOF) angiography,^14^ in this case the increased spatial sampling density of the technique allowed the deconvolution of the slice profiles for super‐resolution reconstruction in the slice direction. Here we focus on how continuous RF shifting produces a moving front of excitation with a single continuous stable signal state. This results in an efficient method for acquiring data with dense sampling in the temporal and spatial domains for free‐breathing abdominal imaging.

## 2 METHODS

### 2.1 Continuous stable state acquisition

Conventional MRI slice acquisition is performed by the application of an RF excitation pulse in combination with a slice selecting magnetic field gradient (*G*_*ss*_). The frequency (ν) required to excite a slice location *z* relative to isocenter is described by:

(1) $$\nu \left(z\right)=\gamma \left(B_{0}+G_{\mathrm{ss}}z\right),$$

where γ is the gyromagnetic ratio and *B*_0_ is the main magnetic field strength.^15^ Conventional M2D imaging methods excite at a single frequency for each location until all the data needed for a complete image is obtained, then a step change in frequency is applied, so the function ν(*z*) recorded for all pulses in an entire acquisition resembles a staircase. We apply an alternative approach where the excitation frequency of each consecutive RF pulse is shifted according to Equation 1 such that each new pulse excites a slightly shifted slice profile forming a slowly moving, localized excitation that travels across the volume of interest.

We hypothesize that if the per TR increment δ*z* in position *z* is small, a stable magnetization configuration can be achieved. This would obviate the requirement for dummy start‐up pulses at the start of each slice position since the magnetization state is spatially and temporally continuous across the slice direction. The approach capitalizes on the softer slice profiles produced by short RF pulses typically deployed in short TR sequences. The leading edge of the slice profile provides a slow ramp up of flip angles as the excitation region sweeps across the imaging volume. The result is that a section of tissue is brought into a gradually evolving magnetization trajectory, experiencing what are effectively a series of catalyzation pulses as the slice sweeps across the tissue. Since the slice profile is continuously shifting, the formal definition of a steady state is never reached. However, after a period the magnetization profile across the slice settles, producing stable signals, and we refer to this as a continuous stable state of magnetization.

The speed at which the pulse profile travels across the FOV therefore determines the closeness between the achieved stable state and a static steady state. The slice profile velocity *v*_*ex*_ can be described by *v*_*ex*_ = δ*z*/TR. For M2D imaging δ*z* would be 0 everywhere except the first pulse in a slice. For SWEEP the rate (*R*_*s*_) at which the excited profile moves across the imaging volume is conveniently defined as the percentage of the full width at half maximum slice thickness (Δ*z*) that is shifted with each consecutive pulse *R*_*s*_ = (δ*z*/Δ*z*) × 100. Defining the sweep rate in this way reflects the fact that a thicker slice can shift larger absolute distances with each pulse than a thin slice without creating unstable signal.

The SWEEP method excites a range of tissue locations and for a given slice thickness Δ*z* the effective slice thickness Δ*z*_*s*_ will be higher. For a given profile velocity a SWEEP slice contains information from the sum of the stable state slice thickness and the additional distance the profile travels over the duration of a slice data acquisition:

(2) $$\Delta z_{s}=\Delta z+v_{\mathrm{ex}}N_{\mathrm{pe}}\text{TR},$$

where *N*_*pe*_ is the number of phase encodes per slice.

### 2.2 Simulations

Simulations were performed to assess the impact of the SWEEP approach on the excited signal under the effect of through plane motion and flow. An open repository of source code for the simulations can be found at https://github.com/mriphysics/sweep-simulation. Magnetization evolution was modeled using a discrete element tissue model based around the extended phase graph (EPG) method^16^, ^17^, ^18^ as implemented by Malik et al^19^. The simulations used vendor optimized short RF pulses: a symmetric truncated sinc‐Gauss pulse for balanced sequences and an asymmetric sinc‐Gauss pulse for RF spoiled sequences. The excited slice profile was applied across simulated tissue with length *L* and specified values for T_1_ and T_2_. The problem was formulated as a time series of flip angles such that each element of tissue experiences a vector of flip angles of length equal to the total number of pulses *α*_*z*_(*t*). These vectors were concatenated along *z* to define a matrix of flip angles *F*(*z*, *t*) = [α_1_(*t*), α_2_(*t*)…α_*L*_(*t*)] describing the series of flips experienced at every element of tissue. The EPG simulation was then performed for each column of *F* resulting in a temporally and spatially resolved model of the signal produced, *S*(*z*, *t*).

The simulation framework was extended to include the effects of through slice motion to allow the inclusion of respiration and flow effects. Respiratory motion was modeled by modulating *F*(*z*, *t*) with a sinusoidal pattern in its temporal dimension and flow was simulated by adding a constant displacement per TR. For respiration, the amplitude of through plane displacement was 4 mm (±2 mm), chosen to be consistent with the medial‐lateral displacement in sagittal acquisitions.^20^

Three simulation studies are presented (see Table 1 for relevant parameters):

**Table 1:** Summary of simulation parameters

Simulation	Sequence	T_1_/T_2_ (ms)(tissue)	Δ*z*	α[∘]	TR (ms)	*R*_*s*_(%)	Respiration	Flow (mm/s)
1: Stability	bSSFP	1412/50 (SM)	4	22	6	0/0.14	‐	‐
	SPGR			5	15			
2: Respiration	bSSFP	1412/50 (SM)	4	21	6	0:1	±2 mm at 0.3 Hz	‐
3: Flow	SPGR	1550/275 (AB)	3	70	15	0:1	‐	−40:40
		1820/99 (GM)						0

Motivation for each simulation is described in text. Abbreviations: AB, Arterial blood; GM, Gray matter; SM, Skeletal muscle.

1. to demonstrate the effect of the SWEEP method on the stability of slice signals in dynamic imaging. Here, dynamic imaging refers to sampling approximately the same slice location repeatedly to capture a time varying process. In the example presented there are five slices and three dynamics (repeats), with four acquisition orders compared. The first scheme interleaved slices and dynamics to maximize recovery between slices, as would be performed for ssFSE and EPI. The second scheme used an ascending slice order with interleaved dynamics, in a way that minimizes the temporal separation of dynamics. The third slice scheme replaced the dynamics with dense equally spaced slices with a high degree of overlap. The fourth scheme utilized the proposed SWEEP method that keeps sampling temporally close and increases spatial sampling density.
2. to investigate the effect of the SWEEP method and different sweep rates on the slice profile in static and moving tissue. The method was demonstrated with two 2D sequences, a balanced steady state free precession (bSSFP) with {0, *π*} phase cycling and a spoiled gradient echo (SPGR) with 150^∘^ phase increment. The simulation began with 500 TR cycles to initialize a stable signal state before modeling the effects of motion with a sinusoidal displacement of underlying tissue.
3. to investigate vessel contrast in inflow angiography. In this case a two compartment simulation was performed with separate static tissue and flowing blood models and the ratio of the signal produced by each used to quantify inflow contrast. In this case the sweep rate was explored linearly between *R*_*s*_ = 0−1%. For comparison a typical M2D (*Rs* = 0%) acquisition scheme was simulated with a new slice started every 90 RF pulses.

### 2.3 MRI methods

A series of MRI imaging experiments were performed on a 3T clinical system (Achieva, Philips Healthcare, Best, Netherlands) to validate the SWEEP technique. A summary of relevant acquisition parameters is given in Table 2.

**Table 2:** Summary of acquisition parameters. Motivation for each acquisition is described in text

Acquisition	Δ*z* (mm)	*R*_*s*_ (%)	Δ*z*_*S*_ (mm)	FOV (mm)	Resolution (mm)	#slices	TR/TE (ms)	Acceleration	α[∘]	Acq. Dur (s)
1: Brain anatomical (bSSFP)	3.0	0.05, 1.45	3.09, 6.00	320 × 320	1.0 × 1.0	10‐256	6.8/3.4	SENSE = 3	80	3‐81
								PF = 0.6		
2: Uterine anatomical (bSSFP)	4.0	0.17	4.40	280 × 332	1.3 × 1.3	550	7.3/3.6	SENSE = 2.2	80	240
								PF = 0.6		
3: Uterine angiography (SPGR)	3.5	0.17	3.80	280 × 332	1.1 × 1.1	630	11.0/6.2	SENSE = 3	50	395
								PF = 0.6		

Abbreviations: Δ*z*_*S*_, effective SWEEP slice thickness; Δ*z*, Nominal slice thickness PF, Partial Fourier; SENSE, Sensitivity encoding.

The first SWEEP validation experiment (Table 2, acq. 1) was a comparison of varying sweep rates on image contrast. This experiment was performed on the brain since it provides a good example of in vivo tissue without large motion effects. SWEEP scans were performed with *R*_*s*_ = 0.05 and 1.45%, and compared to conventional M2D acquisitions, where total slice coverage was matched and the number of RF excitations excluding dummy cycles were the same in both. In cases where SWEEP acquisitions are compared to conventional M2D the parameters for each acquisition are identical, except that the RF frequency does not change over a slice acquisition for M2D (i.e. *Rs* = 0%). The slice acquisition order for M2D was ascending (see Figure 2C, G) and vendor default start‐up pulses were deployed before each slice.

The main application we envisage for the described method is improving the efficiency and signal stability in dynamic M2D acquisitions. We applied the method to free‐breathing respiration resolved abdominal imaging, where spatially dense and efficient M2D acquisitions can be retrospectively sorted to produce 4D (3D + respiratory phase) volumes with high motion tolerance. In this scenario, there is a requirement that the sweep rate is slow enough that the excitation profile travels at most one nominal slice thickness within each respiration cycle. Data were obtained using both bSSFP and SPGR sequences for anatomical and angiography information, respectively (Table 2, acq. 2 and 3). These acquisitions were performed in free‐breathing pregnant subjects as an example of imaging in the presence of complex motion. Informed consent was obtained from 10 pregnant volunteers (gestational ages: 23‐36 weeks) who were scanned in the supine position with routine blood pressure and pulse oximetry monitoring.^21^ No sedation or gating methods were used. In the case of fetal/placental imaging the maximum gradient slew rates were constrained which resulted in measured acoustic noise generated by the sequence of less than 108 dBA.

### 2.4 Resolving respiration

To demonstrate one potential use of the SWEEP technique a simple method is presented for sorting the resulting spatiotemporally dense data into respiration resolved volumes of abdominal organs. All post‐acquisition methods were implemented in Matlab (R2018b, Mathworks, Natick, Massachusetts).

**Figure 1:**
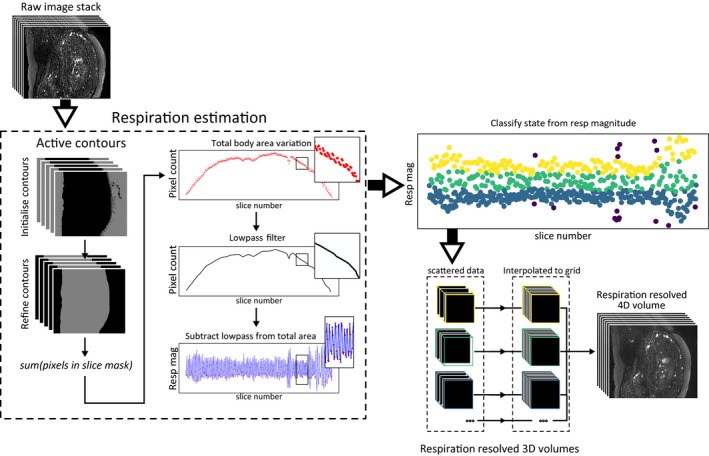
Respiratory position was estimated based on automatic segmentation of maternal body area. A region mask was defined and the maternal boundaries segmented based on an active contour model. The number of pixels within the maternal boundaries in each slice mask defined the total body area within each slice. A low‐pass filter was then applied to remove body area variation unrelated to respiration. The result is a time series which can be used as a measure of respiration. Each slice is then classified into respiratory states based on the local magnitude of this respiration signal. Each respiration resolved stack was then resampled into a full 4D volume

Respiratory sorting requires a measure of the respiratory position, which was estimated here from a slice‐by‐slice automatic segmentation of the patient body area (see Figure 1). Broadly, a rectangular region of interest was manually defined across the body in a region with clear respiratory motion between slices. Tissue‐air interfaces were roughly estimated with a manually set low intensity threshold and refined with an iterative Chan‐Vese active contour model.^22^ The sum of pixels within each slice of the resulting mask of maternal boundaries provides a direct measure of the size of the mask in each slice. This quantity contains information both about respiration, but is also changes with the evolving anatomical content as the slice moves across the volume. Since the latter component varies slowly relative to the respiratory signal a moving average filter was applied and result subtracted off. The final numerical values were deployed as surrogate respiration measures.

The local magnitude of the respiration measure was then used to bin images into *N* states. Here “local” was empirically defined as all slices within 15% of the total number of slices in the time series produced by SWEEP for each slice. Any slices with a respiratory measure outside of 1.8× standard deviation from the local mean were excluded as outliers. Following this classification the SWEEP time series of images could be separated into *N*× 3D stacks. Each individual stack had uniform pixel spacing in‐plane and scattered slice locations in the z direction. To create 4D data the stacks were resampled into a regular 3D grid with isotropic voxel spacing using natural neighbor interpolation, an efficient 3D scattered interpolation method with similar results to cubic interpolation.^23^ The output of this pipeline is a 4D data set with three isotropic resolution spatial and one respiratory resolved dimension.

## 3 RESULTS

### 3.1 Simulations

**Figure 2:**
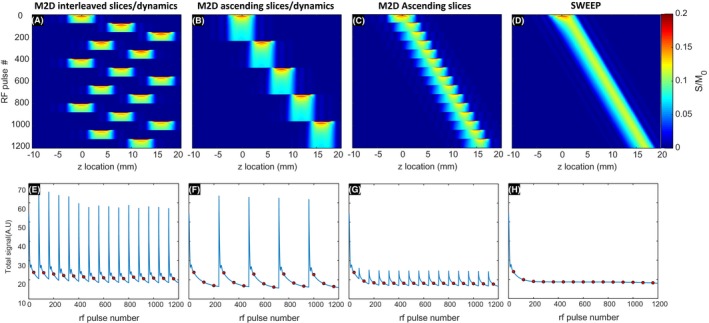
Comparison of M2D bSSFP acquisition schemes all with the same total coverage with data sufficient for the same number of images in same total time. The top row shows the signal matrix *S*(*z*, *t*) and the bottom row shows measured signal as a function of RF pulse. A new phase encoded line of data is acquired after each RF pulse and markers indicate the center of each 2D k‐space. The interleaved order (A and E) shows consistent signal but varies greatly over the slice duration. The ascending slice order (C and G) shows consistent signal between slices but varies over dynamics. The SWEEP order (D and H) produces stable signal between slices and dynamics following the initial transient at the start of acquisition

The results from the simulations investigating signal stability in dynamic imaging can be seen in Figure 2. All acquisition schemes acquire an equal number of 2D k‐spaces with one phase encoded line of data acquired after each RF pulse, but changing acquisition ordering has implications for signal magnitude and stability. These simulations make no assumptions about the sampling scheme and so are applicable to a wide variety of 2D k‐space trajectories. In Figure 2E‐H the center of k‐space for linear phase encode order Cartesian sampling is marked to indicate the influence of unstable signal across multiple consecutive slices.

The interleaved order (Figure 2A,E) rapidly settles to consistent signal between slices and the large temporal separation of dynamics allows for close to full longitudinal recovery between acquisitions. However, the intra‐slice signal varies greatly during the per frame approach to the steady state. This can lead to image artifacts due to irregularities in signal across k‐space. The large temporal separation of samples at the same spatial location would lead to discontinuities in slice content between dynamics if there is tissue motion between slice repeats, making this scheme inappropriate for resolving the respiratory component in free‐breathing imaging.

In the case of an ascending slice order with sequential dynamics (Figure 2B,F), all dynamics for a given slice are temporally close. This ensures dense sampling in time for each slice, but leads to signals that change between images in a dynamic series. The signal within acquired images become more stable later in each dynamic time series as a steady state is approached, but each time the slice location increments a new transient is initiated, leading to repeated variations in signal.

The dense ascending slices acquisition (Figure 2C,G) simply spreads the total number of slices to be acquired uniformly of the region to be imaged. This means that although every location is sampled only once, the high degree of overlap between slices provides temporal information for each local region. In this way, dynamics can be considered as separate slices with a high degree of overlap. These densely sampled slices are temporally close but this scheme retains an unstable transient signal at the start of each new slice. In the case of SWEEP (Figure 2D,H), the sampling is dense with temporal information available for each local region as before, but since the slice profile moves at a constant rate the signal becomes stable. This removes the characteristic fluctuations seen in the other three sampling schemes creating an acquisition that produces stable signal and dense sampling in the sense that each acquired image is close in both space and time to its neighbors.

**Figure 3:**
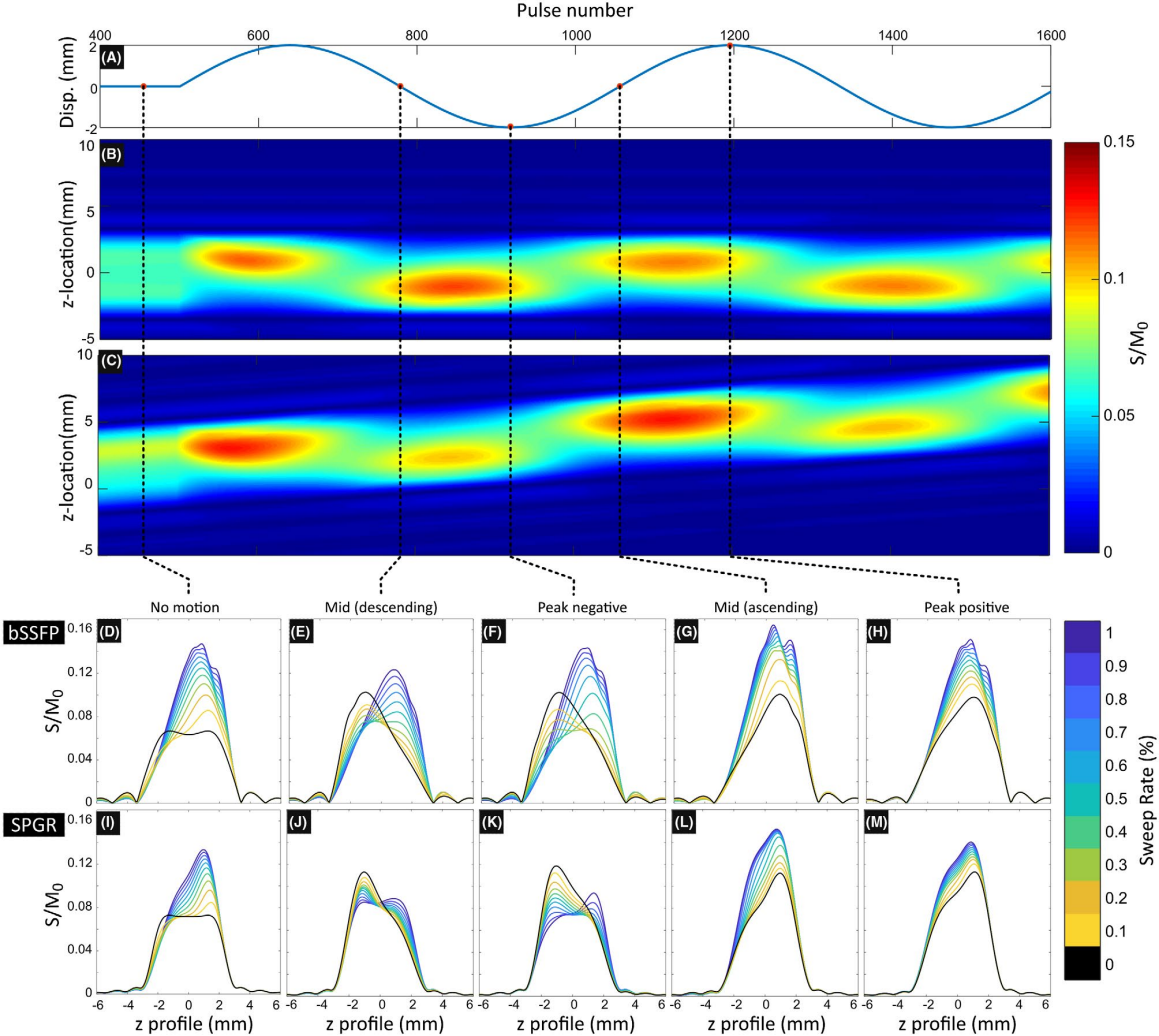
Simulation demonstrating the effect of respiration (A) on the steady state slice profile produced by bSSFP (D‐H) and SPGR (I‐M) acquisitions with a range of sweep rates. Full signal matrices *S*(*z*, *t*) are shown for sweep rates of 0% (M2D) (B) and 0.1% (SWEEP) (C) for the bSSFP acquisition, signal is quantified as the fraction of measured signal to equilibrium magnetization (*S*/*M*_0_). The distortions to the slice profile created by the SWEEP method and induced by normal physiological motion for an M2D excitation are of the same order. This can be seen in detail in the 2D slice profiles produced by a range of sweep rates (color) and a conventional M2D acquisition (black). The effect is also seen in SPGR acquisitions to a lesser effect due to the rapid onset of the steady state in this case

The results from the second set of simulations, performed to investigate how the SWEEP slice profile is affected by sweep rate and underlying tissue movement such as from respiration are shown in Figure 3. A stable signal state was achieved for each sweep rate before the onset of sinusoidal displacements (Figure 3A) to investigate how the sweep rate and respiration interact. Respiration here is described by a through plane displacement, where positive displacement moves in the same direction as the excited profile sweep. The resulting signal matrix *S*(*z*, *t*) is shown for *R*_*s*_ = 0% (M2D) and 0.1% (SWEEP) (Figure 3B and C, respectively). These figures show the signal produced from each position in space with the applied RF pulse series. The signal variation along lines drawn vertically across these matrices show the 1D slice profile at that point in space/time. Example profiles are shown with sweep rates from 0‐1% for bSSFP (Figure 3D‐H) and SPGR (Figure 3I‐M) acquisitions in static tissue (D and I), mid respiration (exhale (E and J)), peak expiration (F and K), mid respiration (inhale (G and L)), and full inspiration (H and M). The simulations in Figure 3 makes no assumption about the number of phase encodes required to fill one 2D k‐space. In practice this simulated series of slice profiles would consist of many individual slices. Effectively this would split the *x*‐axis into linearly spaced chunks of *N*_*pe*_ RF pulses, each chunk would then constitute a single 2D image.

The slice profiles in the absence of tissue motion or sweeping (*R*_*s*_ = 0) display a typical steady state profile that is symmetric across the slice (Figure 3D,I, black contours). In the SWEEP cases we see the profile produced is skewed toward the leading edge of the sweeping slice, with the degree of skewness related the sweep rate. Once the sinusoidal motion is applied the M2D case (*R*_*s*_ = 0) also becomes skewed and more similar to the SWEEP case (Figure 3E‐H,J‐M, black contours). The underlying mechanism generating this behavior is the same; movement of fresh tissue into the excitation profile introduces a component of the slice that has not been subject to the repeated excitations and thus not yet in a saturated signal state. Consequently there is higher signal close to the edge where the new tissue enters. There is a mirrored effect when the displacement is positive or negative (see Figure 3E,J and G,L). Note that whether this new tissue is brought in through the motion of the slice profile or of the underlying tissue is irrelevant. The result is that the difference between the profiles produced by conventional M2D excitation and SWEEP are similar under normal physiological motion at slow sweep rates. Note that this skewness results in a thinner FWHM slice profile when SWEEP or physiological motion are present. This means that Δ*z* decreases and partially offsets the effect of SWEEP producing thicker slices (Equation 2), this is demonstrated in supplementary Figure S1. At fast sweep rates the pulse profile can be become unstable, this is best seen by the jagged bSSFP slice profiles at *R*_*s*_ = 1.0% (Figure 3D‐H). This instability is a potential limitation of bSSFP in some applications and applies also to M2D methods under certain physiological conditions but is not much exacerbated by SWEEP at slow sweep rates. The simulations shown in Figure 3 describe the case of smooth respiratory‐like motion, in the case of a rapid short duration through plane displacement such as can occur in fetal motion or due to coughing during general abdominal imaging, the stable signal state is temporarily broken before becoming re‐established once the motion decreases or stops (Supporting Information Video S1). The time taken for the magnetization to return to its pre‐motion stable signal state is similar for M2D and SWEEP, suggesting that SWEEP is not noticeably more susceptible to adverse effects from unpredictable motion. In cases where sudden or large motion patterns are expected, the SWEEP method can be perhaps best be deployed using slower sweep rates. With dense slice sampling, if sudden motion corrupts a given slice, the surrounding slices are likely to contain a significant portion of information from the corrupted slice location, providing data redundancy. Note also that the initial transient approach to the steady state results in higher magnitude and longer duration oscillations than even the largest simulated motion. Although both SWEEP and M2D are susceptible to sudden motion SWEEP only needs to enter the steady state once whereas M2D repeats this transient at the start of every new slice location.

**Figure 4:**
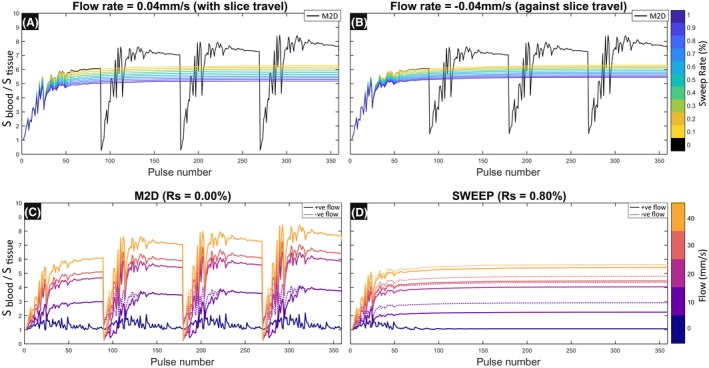
Inflow contrast with SWEEP has some dependence on the direction of flow, dashed lines indicate flow with a negative velocity, solid lines have positive velocity, each defined relative to sweep direction. Contrast is defined as the ratio of signals produced by blood and tissue (*S*_blood_/*S*_tissue_) When the flow travels in the same direction as SWEEP (A) the stable state produces slightly less contrast to the equivalent sweep rate with flow against the sweep direction (B). There is also a large difference in signal stability between M2D (*R*_*s*_ = 0) acquisitions and SWEEP (*R*_*s*_ > 0). The start of each slice produces periods of large signal variations in the tissue and blood components resulting in variable inflow contrast over a range of flow speeds (C). When using the SWEEP method with equivalent coverage (*R*_*s*_ = 0.80% (D)) the contrast is stable for each flow speed over the duration of the acquisition. Dashed lines indicate flow with a negative velocity, filled lines have positive velocity (relative to sweep direction)

The SWEEP method constantly brings fresh unperturbed spins into the slice profile over the duration of a slice acquisition, which is the same mechanism by which inflow contrast works. In TOF angiography the fresh spins are brought by flowing blood, and so only localized regions display inflow enhancement, in SWEEP the entire slice produces a low level enhancement effect. The effect of sweep rate and flow speed on inflow contrast for a 2D angiography sequence is shown in Figure 4. This simulation illustrates the inefficiency of the conventional M2D (*R*_*s*_ = 0) acquisition schemes which produce large transient variation at the start of each new slice (black lines in Figure 4A,B). These transient signals produce variable inflow contrast over the duration of a slice acquisition and may cause image artifacts. The SWEEP method (*R*_*s*_ > 0, colored lines in Figure 4A,B) does not induce such transients as the excitation location moves smoothly across the volume. This causes higher signal than usual from background tissue, which is intentionally being saturated, causing a reduction in the inflow contrast of angiography scans. This means that the degree of background suppression is tuned by sweep rate, with faster sweep rates producing less saturation in static tissue.

The M2D acquisition produces highly variable inflow contrast for different flow velocities (Figure 4C). The SWEEP method produces a stable signal depending only on the flow speed, with slightly reduced contrast when the flow velocity is in the same direction to *v*_*ex*_ (Figure 4D (dotted lines)). This is due to the reduced signal from blood component as a smaller fraction of new spins are entering the slice between each TR when these directions are the same. It is notable that positive and negative flow are also different in the M2D case in this simulation (Figure 4C (dotted lines)). This is caused by the flowing blood's residual signal in the space immediately following successive slices, this can cause interference with the measured signal depending on whether the flow is into or out of the neighboring slice. When flow is fast relative the slice thickness, the direction of blood flow has little effect on measured signal in M2D imaging. However, blood flowing slowly in a negative direction may be subjected to multiple RF excitations in two slice locations which results in a transient signal influenced by the previous slice. In the examples presented, this is most clearly seen for the 10 mm/s case (Figure 4C).

### 3.2 bSSFP anatomical imaging

**Figure 5:**
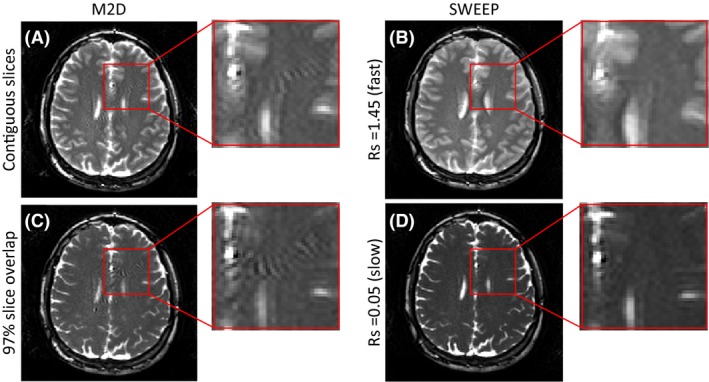
Comparison of M2D and SWEEP excitation acquisitions which acquire an equal slice number and coverage demonstrating the SWEEP stable state. (A) shows M2D with contiguous slices, showing pronounced artifacts originating from the transient approach to the steady state. (B) the continuous stable signal from the SWEEP approach effectively suppresses these artifacts. When a high degree of slice overlap/slow sweep rate is used the images take on T_2_/T_1_ contrast. The M2D acquisition (C) still produces transient artifacts despite the high slice overlap while the SWEEP images produce stable state T_2_/T_1_ contrast(D). Inset images show zoomed views highlighting artifacts in the M2D that are not present with SWEEP

Although bSSFP does not produce much contrast between gray and white matter in the brain due to their similar T_2_/T_1_ ratios, neuroimaging provides an effective *in vivo* setting in which signal evolution in conventional M2D and SWEEP methods can be explored in the absence of significant tissue motion (Figure 5). A comparison is made between M2D scans using the ascending slices scheme (e.g. Figure 2C) and SWEEP scans with the equivalent *R*_*s*_ that require the same number of RF pulses (excluding dummy cycles) to cover the same volume and produce the same number of images. This creates an intuitive point of comparison, but makes the SWEEP acquisition slightly more efficient (by 8% in this case compared to vendor standard start‐up parameters).

Figure 5 demonstrates the improved signal stability with the SWEEP method compared to M2D. In each of the M2D slices image artifacts can be seen as a rippling intensity in several locations in the brain tissue (Figure 5A,C). The origin of these artifacts is the fluctuating signals in the approach to the steady state at the start of each slice. When contiguous M2D slices are used (Figure 5A) images have relatively T_2_ weighted contrast, seen in the grey/white matter contrast, this is due to the the number of RF pulses being insufficient to reach a steady state. The equivalent SWEEP acquisition (Figure 5B) also shows relatively high T_2_‐contrast, in this case this is due to the relatively high fraction of new tissue within each successive pulse profile. However, the rippling artifacts caused by the sudden motion of the slice profile are absent with SWEEP as the signal is consistently maintained in a stable signal state. In the case of a high slice overlap M2D acquisition (Figure 5C) the images are more T_2_/T_1_ weighted as seen by the absence of grey/white matter contrast, however the unstable transient artifacts are still produced despite adjacent slices sharing 97% of excited tissue. The equivalent SWEEP acquisition (Figure 5D) effectively suppresses these artifacts and maintains stable T_2_/T_1_ contrast. Note that the contrast is never identical for M2D and SWEEP even with extreme slice overlap. Similar artifacts can be produced by pulsatile flow near in regions near vessels, however since they are suppressed by the SWEEP method the origin of these artifacts is most likely to be oscillatory signal from tissue approaching the steady state.

Figure 5 provides a good example of the slice thickening effect described above (Equation 2). At the faster sweep rate of 1.45% (Figure 5B) the effective slice thickness is twice the nominal thickness of 3mm. The result is a loss of through plane resolution that manifests as softer gray/white matter boundaries and thickening of brain structures when compared to M2D (Figure 5A). At the slower sweep rate of 0.05% (Figure 5D) the effective slice thickness is only slightly increased over the M2D case (Figure 5C) and the loss of slice resolution is imperceptible.

**Figure 6:**
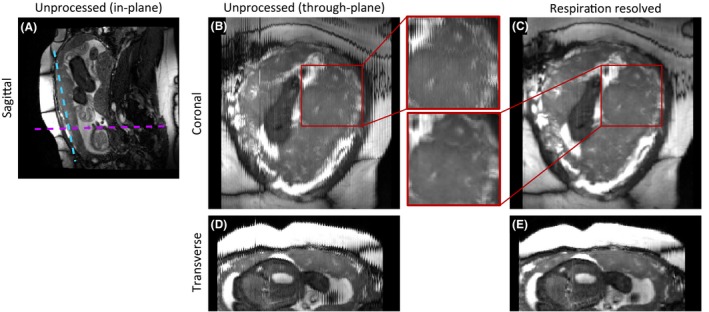
Example 3D respiration resolved images from SWEEP acquisition applied to placenta imaging (GA:28w), an acquisition plane image (A) and before/after examples are shown from a coronal (blue line (A), B,C) and an axial slice (purple line (A), D,E). In orthogonal views before the respiration resolved reconstruction (B,D) there is clear respiratory corruption, especially visible in the bSSFP banding artifacts and on the anterior surface(D). After processing the jagged appearance has been successfully resolved to a single respiration state (C and E). The inset shows a magnified example where the processing has produced a clear improvement in through plane image consistency by removing respiratory artifacts

All acquired pregnant volunteer datasets produced successful respiratory resolved reconstructions. A representative bSSFP respiration resolved dataset (GA: 28.9w) is shown in Figure 6. Pre‐ and post‐respiration resolved images are shown for one sagittal (native plane), and one coronal and axial section through the image stacks. In all planes, the respiration resolved reconstruction removes respiration artifacts and improves visibility of smaller structures. The coronal view (Figure 6B,C) is through the mid‐placenta and shows how respiratory resolved SWEEP can improve visibility of placenta lobules, in particular the bright spots corresponding to faster flowing blood at the spiral artery inlets (magnified in Figure 6J,K). The axial view (Figure 6D,E) shows another view through the same data demonstrating the effective removal of respiratory artifacts.

### 3.3 Inflow angiography

The application of the SWEEP method to TOF angiography produces an efficient and motion robust free‐breathing acquisition. The example given here is applied to uterine angiography where a large field of view, motion and difficulty in achieving breath holds all present problems for typical 3D angiography acquisitions. In these situations, rapid 2D acquisition is required for motion robust imaging at the slice level and retrospective respiratory resolved reconstruction can be used to sort the images into motion corrected 3D volumes.

**Figure 7:**
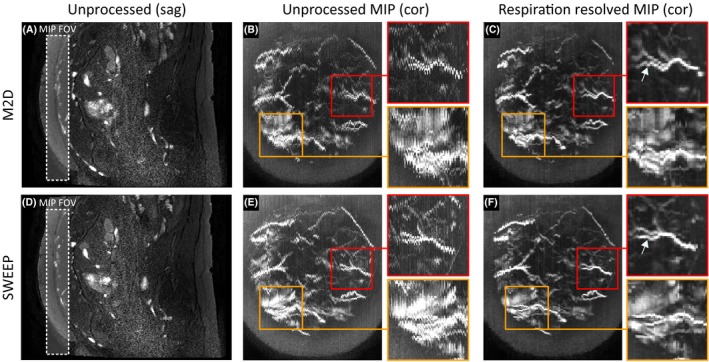
Example SWEEP angiography respiration resolved reconstruction from normal pregnancy (GA: 31w). Unprocessed image data for both the conventional M2D and SWEEP methods are shown on the left (A and D). Comparable MIP views (20 slices) in the orthogonal coronal plane show severe respiratory corruption in the unprocessed image data obscuring vessels (B and E). After respiratory binning and registration methods have been applied the misalignment is reduced improving vessel clarity (C and F). Two example vessels are extracted to demonstrate the improvement made with the SWEEP method. In each example the proposed respiratory sorting method produces good 3D reconstructions of the 2D data. This data suggest that the SWEEP data may provide more smooth and continuous vessel morphology than M2D (arrows) although this is not a clear cut improvement

The unprocessed image data for conventional M2D (Figure 7A,B) and SWEEP acquisitions (Figure 7D,E) show the expected through plane motion corruption, this is best seen in the unprocessed coronal views (Figure 7B,E). Following the proposed processing method for the densely sampled SWEEP data these coronal views become more consistent in the through plane direction (Figure 7C,F). Two vessels are shown in zoomed view to provide comparisons between the conventional M2D and the SWEEP methods.

In the fetal angiography case the benefits of the SWEEP technique, such as homogeneous flow contrast (Figure 4) and reduced transient signal artifacts are secondary to the larger effect of maternal‐fetal motion on image quality. It is difficult therefore to make a direct comparison between the two methods presented in Figure 7. However, based on these images there is evidence that SWEEP may perform better in some respects than M2D. For example, the radial artery (red boxes in Figure 7) shows good improvement from both the SWEEP and M2D data. However, some residual respiratory corruption is visible in the respiration resolved M2D reconstruction that is reduced in the SWEEP reconstruction (Figure 7C,F arrows). A second example (orange boxes in Figure 7) shows another uterine vessel that is obscured by surrounding structures. In the M2D case (Figure 7B,C) the residual respiration effects make it difficult to delineate the vessel from the background, the vessel is clearer in SWEEP acquisition (Figure 7E,F). However, this is likely an effect of reduced motion during the SWEEP acquisition. The apparent change in vessel morphology between the M2D and SWEEP acquisitions (Figure 7C,F) is a consequence of the two scans being performed sequentially. Motion of the fetus, uterine wall and maternal gut can alter the shape the morphology of uterine vessels.

**Figure 8:**
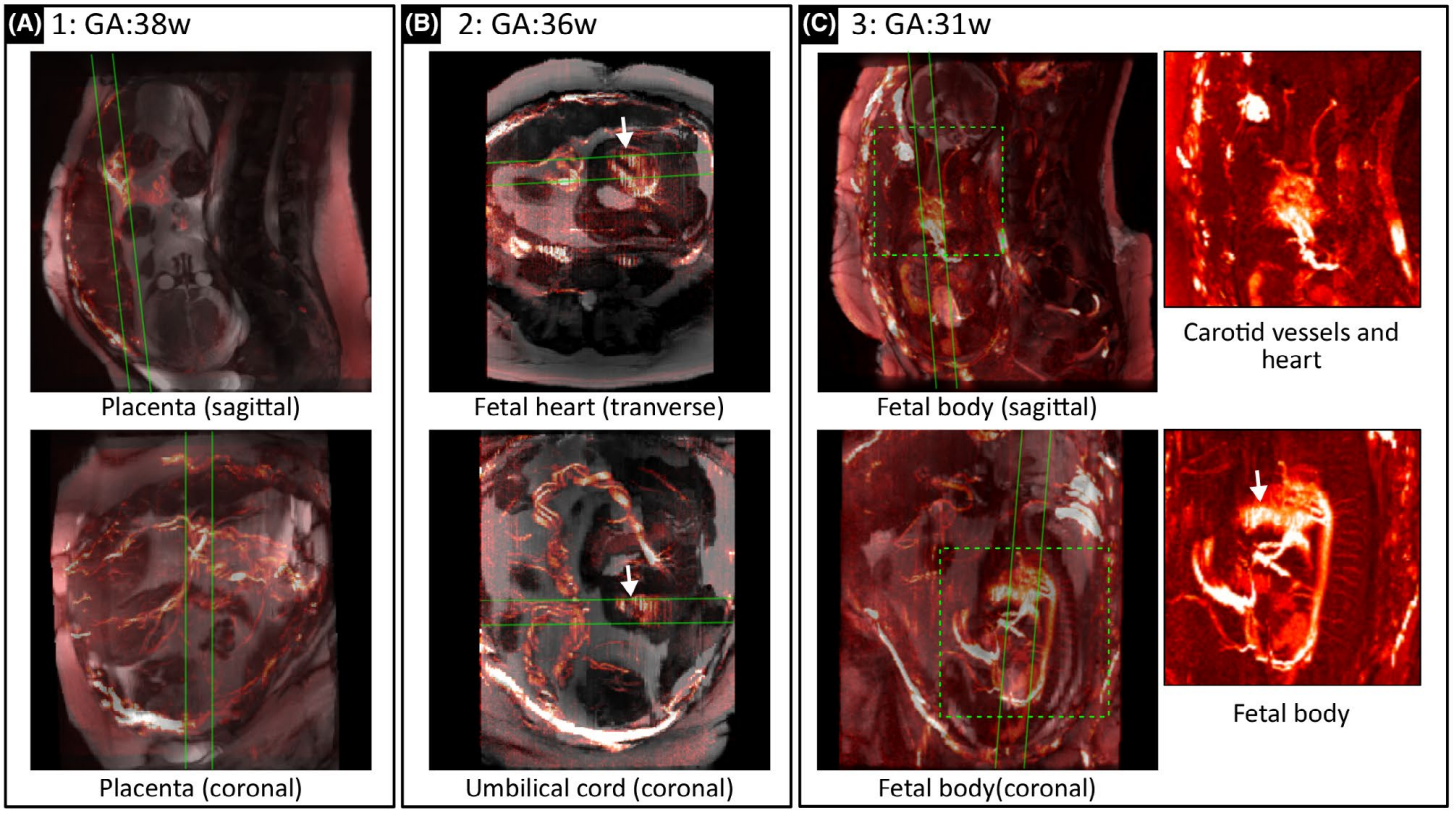
Example SWEEP angiography data and anatomical overlay. Lines define MIP areas in orthogonal views, dashed boxes indicate expanded examples with no anatomical overlay and, arrows indicate artifacts caused by the rapid fetal heart rate. Data from three representative volunteers are shown at GA:38w (A), 36w (B), and 31w (C). In each case, the respiratory corruption has been resolved producing detailed views of fetal/placental vasculature. These overlay images allow identification of fetal/placental features such as the placenta chorionic vessels (A), the fetal heart and umbilical cord (B), fetal carotids vessels and the fetal body (C); including the aorta, femoral arteries, cord insertion, and several medullary arteries

Representative SWEEP angiograms with bSSFP anatomical overlays from three pregnant volunteers are shown in Figure 8. Each image shows a MIP over 20 slices with the boundaries of the MIP plane displayed as green lines in the matched orthogonal image. Data from the first volunteer (GA:38w) (Figure 8A) shows clearly defined placental vasculature on both the maternal uterine wall and chorionic plate. The second volunteer at an earlier gestation (GA:36w) (Figure 8B) shows examples of the fetal heart and umbilical cord. The final volunteer (GA:31w) (Figure 8C) shows well defined fetal vasculature. In this case to facilitate vessel visualization expanded views without an anatomical overlay are shown (locations defined by the dashed green boxes). Note that the fetal heart beats at a faster rate than the slice acquisition time, which results in unstable signal in the heart in the through plane direction causing striping artifacts (see arrows Figure 8B,C).

## 4 DISCUSSION

In this work, we have presented the SWEEP technique as a method to improve the scan efficiency of 2D short‐TR sequences by creating a spatially continuous moving front of excitation that encodes slices while sweeping across an imaging volume. Compared to the conventional M2D approach this method offers a number of advantages, most notably the dense spatiotemporal sampling it offers. Since all sampling is close in space and time data is locally consistent even in the presence of large or unpredictable deformations, such as respiratory or fetal motion, provided individual slice images are fast enough to freeze the motion. This allows detailed reconstruction of large respiratory resolved 3D volumes from SWEEP acquisitions, and in particular allows SWEEP data to be used for respiratory resolved volumetric reconstruction.

A single continuous stable signal state is shown to be more constant than seeking to create a new steady state of magnetization for each slice location. It has previously been demonstrated that although bSSFP imaging is widely considered to be T_2_/T_1_ weighted, it is often in fact a complex combination of proton density and T_2_/T_1_ contrast.^24^ This weighting depends heavily on the number of RF pulses preceding acquisition around the k‐space center and this effect is accentuated as flip angles increase. This affects the limit of acceleration in bSSFP acquisitions since reducing the number of RF pulses through partial acquisition methods means the center of k‐space is then acquired with magnetization further from the steady state. The SWEEP method resolves this by preserving a stable signal state at all times after an initial start‐up period. SWEEP is therefore an efficient method to produce a stronger T_2_/T_1_ weighting in high flip angle bSSFP images without requiring a high number of startup cycles. It should be remembered that there is always contrast variation within each voxel caused by the slice profile due to variable effective flip angle as well as sweeping. As shown in Figure 3, the slice profile is a complex matter that depends on the RF pulse, the tissues, sequence parameters and tissue motion, as well as sweep rate if applied.

The effect of SWEEP on the slice profile is increased signal on the leading edge from unsaturated magnetization entering the slice. The result is that SWEEP produces a narrower FWHM profile than conventional M2D and this slice profile possesses slightly higher signal content than the M2D case (see Supporting Information Figure S1). The SWEEP approach also increases the temporal efficiency of scans by removing the need for dummy cycles during the transient oscillatory magnetization phase for each new slice. There is then only one period of transient signal at the very start of the acquisition. As already noted a number of methods have been presented to reduce the number of RF pulses needed to approach the steady state, but these reduce transients rather than eliminating them. Thus SWEEP can always improve efficiency.

No direct comparison is made here between SWEEP and the various catalyzation schemes available. Catalyzation schemes aim to accelerate the approach to the steady state whereas SWEEP largely eliminates this requirement by creating a stable state that is preserved for the duration of the acquisition. Figure 5 provides a simple comparison to illustrate this, where M2D acquisitions with a simple half‐alpha catalyzation at the start of each slice produce clear artifacts due to the oscillatory signal which are removed by the SWEEP method. Abdominal and free‐breathing M2D and SWEEP acquisitions produce visually similar images since the motion effects of respiration have a greater influence on image quality than the more subtle effect of the preserved stable magnetization state. The key benefit of the SWEEP approach is that it is maximally efficient when producing densely sampled slice acquisitions.

The effect of B1 transmit homogeneity on SWEEP is similar to that of M2D. Regions of inhomogeneity will perturb the stable state as the excitation differs from the nominal flip angle. In the case of SWEEP a single slice may contain different regions of $B_{1}^{+}$ inhomogeneity as the moving slice profile travels across the subject. However, in this study, performed at 3T in pregnant subjects where $B_{1}^{+}$ homogeneity is likely to be a problem, we did not encounter any instances where the SWEEP acquisition was adversely affected.

A key application of the presented SWEEP method is constructing 4D images from stacks of 2D slices in the presence of respiratory motion. This works best when paired with an accurate and robust measure of motion with which to assign motion states to individual slices. For demonstration purposes a simple body area measure was used here, however this does not work well for all image orientations and body area is not directly related to the respiratory position. Refinement of the motion estimate technique may improve performance. An alternative approach to the respiration resolved volume acquisitions described here is to use a respiration gated acquisition. There are a large number of techniques for abdominal respiratory‐gated and navigator based volumetric imaging methods are often application dependent, with no one widely adopted method.^25^, ^26^ Selecting and then optimizing any one of these methods to provide a fair direct comparison with SWEEP is a non‐trivial task, particularly for fetal/placental imaging as these are not standard applications, and has not been attempted. Alternative approaches to image‐based motion estimation such as image correlation^13^ and manifold learning^27^, ^28^ or hardware‐based self gating methods such as coil scattering^29^ and noise navigators^30^ may prove beneficial.

The SWEEP method modifies a basic but fundamental property of MRI acquisition and offers value to short TR imaging methods. The low level nature of this modification means that the method is compatible with a wide range of existing acquisition techniques. The imaging experiment described in this paper utilizes a Cartesian acquisition since this offers the most efficient k‐space filling and is least prone to reconstruction artifacts. Non‐Cartesian trajectories in combination with SWEEP offer some interesting prospects, such as self‐gating signals and compatibility with diffuse sparse reconstruction methods. This could be used to reduce the time per slice and increase the temporal resolution of the respiration resolved reconstruction. For example golden angle radial SPGR with continuous RF shifting produces a helical trajectory that combined with a sliding window reconstruction produces high quality 3D angiography from 2D acquisitions.^14^ A case of where SWEEP is unlikely to offer much advantage is in combination with magnetization prepared sequences such as initiated with inversion and saturation pulses. Magnetization preparation intrinsically seeks to exploit non‐equilibrium magnetization conditions in which there is clear evolution with time. This does not marry with the aim of SWEEP to exploit stable signal states, so that it is not clear how the properties of SWEEP could best be exploited in this type of regime.

Future improvements to the method might focus on aspects that are unique to SWEEP. One example might be designing bespoke RF pulses with optimized excitation profiles, where the edges and side‐lobes of the slice ramp the tissue in such a way as to catalyze a stable state. Here the nominal sweep rate used for excitation was a fixed value. In reality, the motion of the patient means that the true sweep rate is the sum of patient motion and *v*_*ex*_. Introducing a dynamically varying *v*_*ex*_, where this sum is constant would improve signal stability and may have beneficial sampling properties. Since the position of the each acquired signal is spatially continuous in the slice direction the SWEEP method offers a natural pairing with acceleration techniques based on continuous undersampling such as kt‐SENSE/BLAST and golden‐angle methods.^4^, ^31^, ^32^ Finding methods that allow the data support for reconstruction to slide with the slice would allow maximum flexibility.

## 5 CONCLUSIONS

Rapid acquisition of 2D slices is an effective way to freeze local motion in cases where conventional motion compensation techniques are inadequate. Rapid acquisition gradient echo sequences, both spoiled and balanced, induce the magnetization into a steady state that is the intended image acquisition condition, but there is generally an initial transient onset period which, if sampled, produces image artifacts. When multiple 2D slices are required to cover a volume of interest this transient phase can be a significant fraction of total acquisition time. Here we present a method “SWEEP” in which the slice excitation continuously traverses the acquisition volume producing steady signals throughout. SWEEP is demonstrated as an efficient way of acquiring spatiotemporally dense data from moving subjects and was been shown to result in faster and more robust short TR acquisitions when applied to brain, abdominal, and fetal/placental imaging. The dense data was demonstrated to have potential applications for retrospectively reconstructing 4D respiratory resolved volumes where respiratory corruption was effectively removed from bSSFP anatomical and TOF angiography acquisitions.

## Supporting information


**FIGURE S1** The effect of sweep rate on slice thickness (A, C). The FWHM of the excitation profile (blue) becomes smaller as sweep rate increases, partially offsetting the increase in slice thickness due to sweep travel distance (red) the sum of these two effects represents the measured slice thickness (yellow). As sweep rate increases a higher magnitude signal is measured (B, D) due to fresh, unsaturated tissue entering the excitation profile within each TR

**VIDEO S1** Animation illustrating the effect of sudden motion on the stable signal states produced by bSSFP M2D and a range of sweep rates (0‐1%) using the simulation parameters to Table 1—simulation 2. The top row shows the prescribed motion pattern with a long period of no motion followed by a series of motion blips exponentially increasing in magnitude. The second row shows the slice profiles produced by the sequence and how they are distorted by the motion. The Third row shows the same information on a shorter *y*‐axis scale. After the period of motion ends the slice profile is seen to settle back into the previous stable state prior to motion within roughly the same time frame regardless of M2D or sweep rate
