# Supplementary material for: Respiration resolved imaging with continuous stable state 2D acquisition using linear frequency SWEEP
Source: Magn Reson Med. 2019 Jun 10;82(5):1631–45. doi: 10.1002/mrm.27834 (PMC6682494; doi:10.1002/mrm.27834)
Supplement: Supplementary file 1 — FIGURE S1 The effect of sweep rate on slice thickness (A, C). The FWHM of the excitation profile (blue) becomes smaller as sweep rate increases, partially offsetting the increase in slice thickness due to sweep travel distance (red) the sum of these two effects represents the measured slice thickness (yellow). As sweep rate increases a higher magnitude signal is measured (B, D) due to fresh, unsaturated tissue entering the excitation profile within each TR [file MRM-82-1631-s001.docx]

**Supporting Information**

**Supporting Information Video S1**: Animation illustrating the effect of sudden motion on the stable signal states produced by bSSFP M2D and a range of sweep rates (0-1%) using the simulation parameters to Table 1 - simulation 2. The top row shows the prescribed motion pattern with a long period of no motion followed by a series of motion blips exponentially increasing in magnitude. The second row shows the slice profiles produced by the sequence and how they are distorted by the motion. The Third row shows the same information on a shorter y-axis scale. After the period of motion had ended the slice profile is sent to settle back into the previous stable state prior to motion within roughly the same time frame regardless of M2D or sweep rate. This suggests that rapid or non-periodic motion has the same proportional effect on the magnetization whether the RF profile is sweeping or not.

**
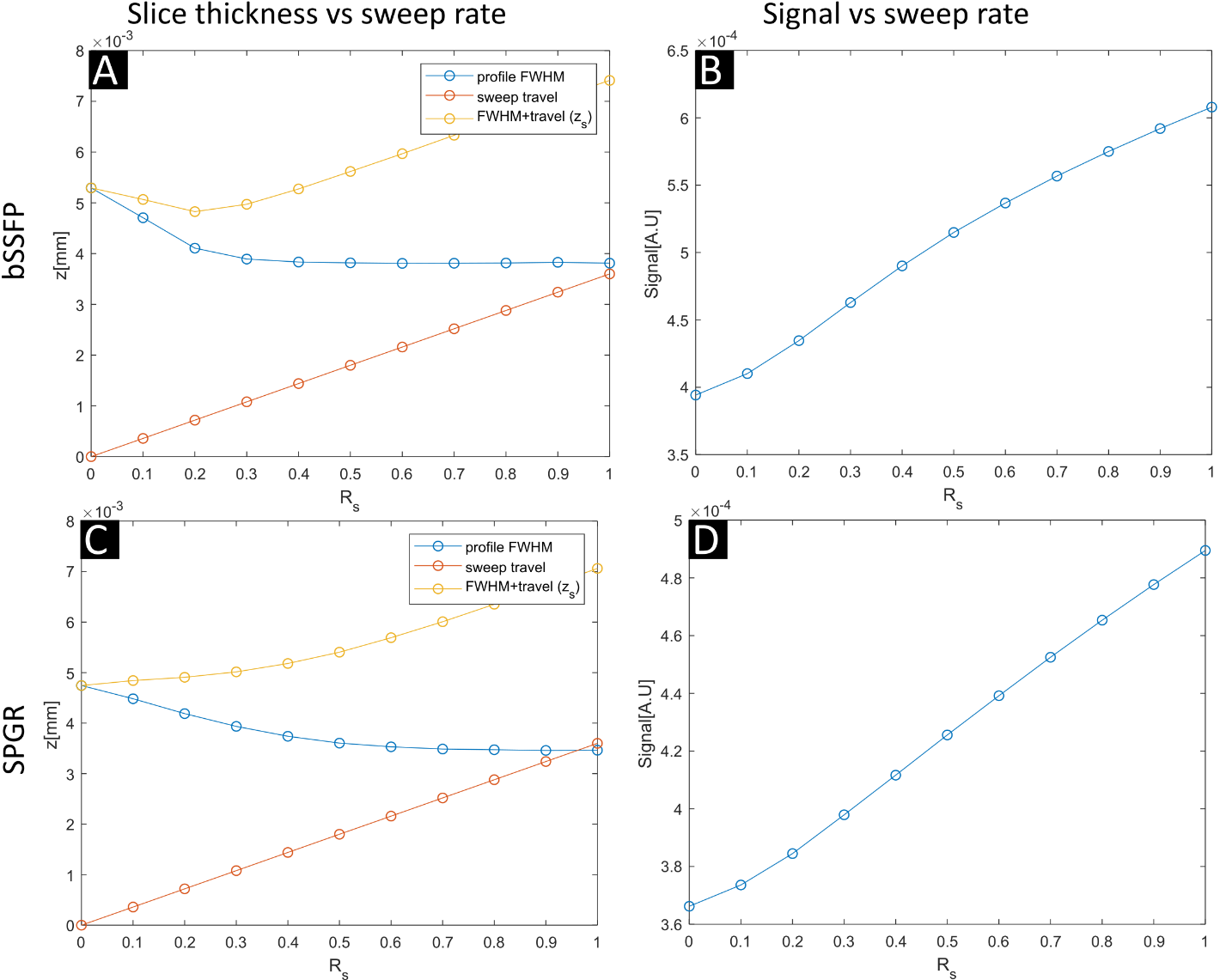
**

**Supporting Information Figure** **S1:** The effect of sweep rate on slice thickness (A, C). The FWHM of the excitation profile (blue) becomes smaller as sweep rate increases, partially offsetting the increase in slice thickness due to sweep travel distance (red) the sum of these two effects represents the measured slice thickness (yellow). As sweep rate increases a higher magnitude signal is measured (B, D) due to fresh, unsaturated tissue entering the excitation profile within each TR.
